# Is There Always a Negative Causality between Human Health and Environmental Degradation? Current Evidence from Rural China

**DOI:** 10.3390/ijerph191710561

**Published:** 2022-08-24

**Authors:** Wei Zhou, Fan Zhang, Shihao Cui, Ke-Chiun Chang

**Affiliations:** 1 College of Public Administration and Law, Hunan Agricultural University, Changsha 410128, China; 2 School of Economics and Management, Wuhan University, Wuhan 430072, China

**Keywords:** One Health, zoonosis, environmental health, sustainable development, integrated governance

## Abstract

This study explores the incidence and trend of zoonoses in China and its relationship with environmental health and proposes suggestions for promoting the long-term sustainable development of human, animal, and environmental systems. The incidence of malaria was selected as the dependent variable, and the consumption of agricultural diesel oil and pesticides and investment in lavatory sanitation improvement in rural areas were selected as independent variables according to the characteristics of nonpoint source pollution and domestic pollution in China’s rural areas. By employing a fixed effects regression model, the results indicated that the use of pesticides was negatively associated with the incidence of malaria, continuous investment in rural toilet improvement, and an increase in economic income can play a positive role in the prevention and control of malaria incidence. Guided by the theory of One Health, this study verifies human, animal, and environmental health as a combination of mutual restriction and influence, discusses the complex causal relationship among the three, and provides evidence for sustainable development and integrated governance.

## 1. Introduction

Coronavirus disease 2019 (COVID-19) has sent shockwaves around the world, and its effects are still rapidly unfolding and will continue. COVID-19 is a zoonotic disease [1] that, like SARS, avian influenza, Ebola, and Zika, has caused a global public health crisis and has jeopardized human health. For now, the risk of zoonoses has remained, with studies revealing that over 60% and 75% of known and emerging infectious diseases are transmitted from animals to humans, respectively [2]. Epidemic outbreaks in recent years have often followed a pattern: a virus was initially passed from animals to humans, and the existence of the new infectious virus was initially not noticed because those infected displayed mild symptoms or those confused with other known diseases. Pathogens seize this opportunity to spread widely in the population, and there is the potential for a “pandemic” situation to develop [3,4,5]. In early March 2020, Finland was forced into a state of emergency when an outbreak of a highly pathogenic avian influenza (H5N8) subtype occurred at the same time as the COVID-19 situation worsened [6]. This raises questions about why society is still vulnerable to emerging infectious diseases. How to reverse passivity, reduce the occurrence and outbreak of zoonotic diseases, improve the defense function of public health systems, build a healthy living environment, and then realize the sustainable development of global public health [7] is a topic that has been of concern for more and more scholars.

Scholars propose to adopt the One Health paradigm to deal with the above problems [8,9,10]. The concept of “One Health” was originally proposed in 1873 and gradually evolved into a cutting-edge concept in the field of public health [11]. The Food and Agriculture Organization of the United Nations (FAO) defines it as the use of a collaborative, international, cross-sectoral, multidisciplinary approach to respond to threats and reduce the risk of infectious diseases at the animal–human–ecosystem intersection. “One Health” provides strategies for a complex situation: integrated governance of human, animal, and environmental health [12]. It is commonly used as a guideline to reduce the risk of human diseases, so as to seek a more harmonious and integrated method to reduce the harm of animal-borne diseases [13].

As a large agricultural country with the largest human–animal interface, China has experienced many emerging animal-to-human epidemics. As its economic and social development has entered a new era, the epidemic and transmission characteristics of animal-to-human diseases have also undergone new changes; new and old problems and difficult problems are intertwined, the prevention and control situation is complicated, and the emergency response to the epidemic has become increasingly difficult [14,15]. The concept of “One Health” provides a perspective to deal with this complex situation, which is to establish an overall view governance of the animal–human–environment, focusing on the interlinkages between human health, environmental health, and animal health [11,12,13].

This study chose China’s rural areas as research objects for three reasons. First, with social development, rural areas have suffered the health consequences of unbalanced economic and environmental development. Rural public health in China is the weakest link in the process of optimizing public health services and health outcomes [16,17]. Health status in rural areas is generally worse than in urban areas, with higher rates of mortality, pain, discomfort, and anxiety [18]. In the past two decades, cancer incidence rates in rural area have been higher than in urban areas [19,20,21]. Income disparities and socioeconomic differences are reasons for this [22]; however, the health benefits of economic growth may have been offset by the effects of environmental degradation [21]. The incidence in rural areas may be related to poverty and chronic infectious diseases [21]. Exposure to carcinogens and differences in sanitary conditions are also important factors [23]. Moreover, as a large breeding country, China’s rural areas are the main battleground for large-scale human–animal contact, and zoonoses occur frequently. Thirdly, the Outline of the People’s Republic of China 14th Five-Year Plan for National Economic and Social Development and Long-Range Objectives for 2035 [24] mention the strategic position of giving priority to the development of a healthy China and put forward the concept of prevention as a priority. Significant changes in sanitary conditions, public health, and other social factors in rural China have been developing in the past two decades [25,26]. Significant data changes can provide good observation samples for this study.

Based on the concept of “One Health”, this study focuses on the complex causality among human, animal, and environmental health, and explores the incidence and trend of zoonoses in various provinces against the background of economic development, as well as the mutual restriction and influence with environmental health. The aim of this study is to explore the relationship between screened independent variables, malaria incidence and environmental factors, climatic factors and economic development, and then to derive countermeasures and suggestions to promote the long-term sustainable development of human, animal, and environmental health systems, and provide a reference for cross-sector integrated governance.

### 1.1. Complex Linkages between Human Health and the Environment

The complex relationship between the occurrence of zoonosis, the human activities, and changes in the natural environment have long been studied [27,28,29,30,31]. There is an objective link between the outbreak and long-term development [29], and the challenges of pandemics are not limited to health but are linked in a broader context.

Perreault, Bridge, and McCarthy [30] argued that the relationship between people and the environment was the result of political influence, whereas the emergence of the pandemic was influenced by political ecology. Wallace [31] suggested that the emergence and spread of diseases do not align with the “patient zero” transmission mode constructed in epidemiology, but in the process of interaction between politics, social relations, and ecology on a space-time scale, while the emergence of pandemic diseases is the result of a series of overlapping structural processes. Therefore, studies on the root causes and prevention of zoonoses need to avoid simplistic, linear, and causal narratives and investigate prevention and response strategies in a new way of thinking and action.

Dzingirai, Bett, Bukachi, Lawson, Mangwanya, Scoones, Waldman, Wilkinson, Leach, and Winnebah [27] discussed differences in infection at the social level. In a study of trypanosomiasis cases in Zimbabwe, people who had more opportunities to come into contact with livestock and the wild environment for work or life had a higher probability of infection. In the case of Lassa fever in Sierra Leone, women were more likely to be infected because they were engaged in work and housework in environments where the virus reservoir was concentrated. Studies have demonstrated that occupation, gender, age, and wealth are all potential determinants of disease infection. This conclusion is consistent with Leach et al. [32], who noted that rapid changes in structural, social, and political ecology have led to non-linear interactions between humans and animals, resulting in uncertainties in disease occurrence and form, which lead to the spread of diseases. For example, the spread of agriculture indirectly changed the transmission pattern of trypanosomiasis in the area; commercial agricultural irrigation in Kenya has expanded the mosquito-borne habitat and, coupled with uncertain climate factors, has contributed to the spread of the disease. The change in political ecology and economic development of aquaculture in the outbreak of avian flu and swine flu has an important role. As such, south-east Asian countries, where the demand for poultry is growing faster, are more affected by the epidemic, and the rapid growth of medium-sized enterprises in the industry have very limited levels of biological safety control is at the core of this [33].

The root causes of diseases are uncontrollable, complex, and uncertain, and the level of understanding is still very limited. Many policies and approaches focus on treating symptoms, resulting in programs and interventions that quickly disintegrate [34]. There are currently still significant uncertainties in the methods of disease tracking. For example, the current main method of scientific advice and prediction for public health events is the application of epidemic models [35], and new perspectives and ways of thinking should be embedded. Only in this way we can have a more accurate understanding of how the disease spreads, its impact, and how to manage it according to local conditions [36].

Rüegg et al. [37] proposes a systems theory-based evaluation framework called NEOH (Network for Evaluation of One Health) to address the inherent complexity of One Health initiatives with a view to improving human, animal, and environmental health. The NEOH assessment framework consists of four overall elements, namely: (a) OH initiative and its context, (b) a description of the theory of change, (c) an OH-ness assessment of different aspects of One Health (thinking, planning, sharing, learning, interdisciplinary, and leadership), and (d) an assessment of the links between the process assessment and the results produced. Based on this, scholars carried out applied research in the field of epidemiology of epidemic diseases [38,39,40,41]. Zoonoses prevention and risk assessment system can be established with a One Health approach [42,43,44].

### 1.2. Zoonoses and Environmental Status of Rural China

**Figure 1:**
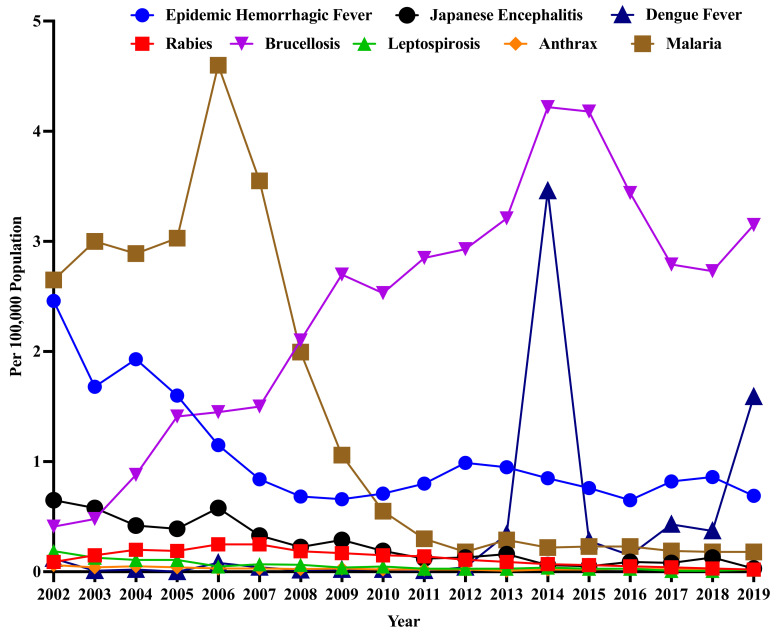
Incidence of zoonoses in China (excluding diseases for which no continuous data are available). Source: China Health Statistical Yearbook (2002–2019).

As of 4 February 2020, there are 40 notifiable infectious diseases, including two Class A and 27 Class B infectious diseases. According to the “List of Zoonotic Diseases” formulated by the Ministry of Agriculture and Rural Affairs of China Notice No. 1149 [45], a total of 26 animal diseases have been included in the list. By filtering out inconsistent data, eight zoonoses were identified: epidemic hemorrhagic fever, rabies, leptospirosis, brucellosis, anthrax, Japanese encephalitis, malaria, and dengue fever. The temporal distribution of the incidence of the eight zoonoses is illustrated in Figure 1.

Based on the characteristics of China’s rural areas, the selection of environmental assessment indicators differs from that of urban areas. When measuring the degree and efficiency of urban environmental pollution, the main index is industrial pollution. Environmental pollution in China’s rural areas is mainly due to agricultural nonpoint source pollution and domestic pollution. In the Opinions on “Comprehensively Push Forward Rural Vitalization and Accelerate the Modernization of Agriculture and Rural Areas” [46] issued by the Chinese government in 2021, several directions of rural environmental governance are clearly highlighted. These include classifying and orderly promotion of rural toilet renovation and sewage treatment improving the mechanism for disposing of rural household garbage and reducing the use of pesticides and diesel fuel. Therefore, the coverage of harmless sanitary toilets, the amount of pollutants discharged from agricultural sewage, the amount of pesticides used, and so on can be used as measures of the quality of the rural environment. A harmless sanitary toilet is relative to the concept of a sanitary toilet; the standard of sanitary toilet is: wall, roof, septic tank does not leak, airtight cover, toilet clean, basically odorless, excrement according to the provisions of the clear out. Sanitary toilets have no special requirements for the disposal of feces. However, feces without harmless treatment is an important factor leading to the occurrence and prevalence of intestinal infectious diseases and mediated diseases in rural areas. As a result, harmless sanitary toilets have been popularized in rural China. On the basis of sanitary toilet, the harmless treatment technology of excrement is added.

Malaria is a natural infectious disease that is transmitted by the bite of an infected Anopheles mosquito. Its spread is linked to exposure to the source and sanitation, as well as seasonal weather. Among the environmental problems in China’s rural areas, the improper treatment of feces and sewage is the most prominent. In recent years, rural residents’ production mode, living habits, livestock raising methods, and environmental pollution have been found to be correlated with the high incidence of cancer [21], higher incidence of zoonoses, and weaker epidemic prevention and control. The occurrence of disease is a combination of many factors, including dietary habits, excessive consumption of fruits and vegetables treated with pesticides, and poor environmental quality [20,47]. However, there is no doubt that the soil and water environments in rural areas of China are polluted. Agricultural sewage is the main source of agricultural nonpoint source pollution [48,49].

Agricultural sewage refers to the sewage and precipitation discharged by agricultural and animal husbandry production, or the water discharged by irrigation water flowing or seeping/leaking through farmland. The large-scale use of chemical fertilizers and pesticides in agriculture has transformed agricultural production activities, which previously had little impact, into the main source of water pollution. Pesticides, pathogens, and other toxic substances in agricultural sewage can pollute drinking water sources and harm human health [50,51]. When spraying pesticides and fertilizer, generally only a small amount adheres or is applied to crops, and the vast majority of the rest in the form of agricultural runoff ends up as residue in the soil and floating in the atmosphere. Through rainfall, this runoff contaminates surface water, resulting in large-scale soil pollution and damage to the natural ecosystem.

**Figure 2:**
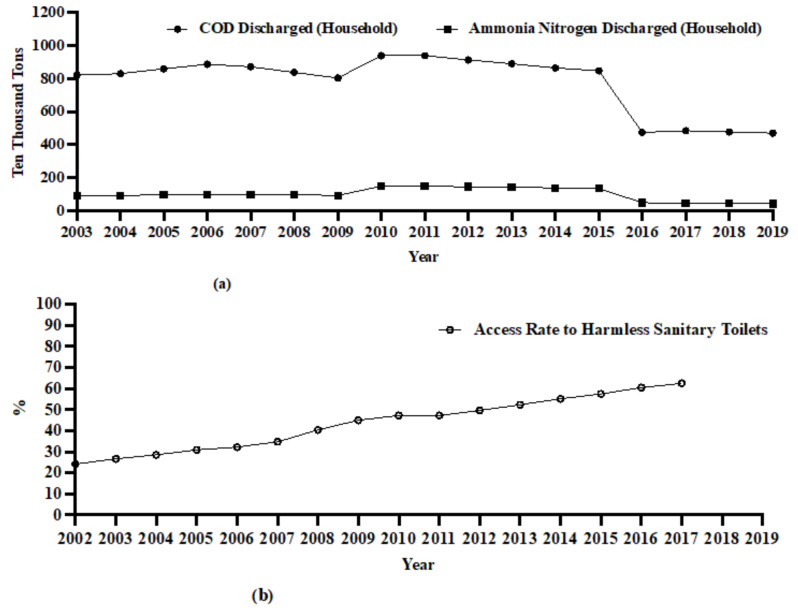
Discharge of wastewater (ten thousand tons) and access rate to harmless sanitary toilets (%). (**a**) COD and Ammonia Nitrogen in sewage discharge (Household). (**b**) Access Rate to Harmless Sanitary Toilets (%). Source: China Environmental Statistical Yearbook (2002–2019).

At the beginning of 2006, the Chinese government proposed “The New Rural Construction Policies” to alleviate and improve the crisis between economic development and environmental degradation in rural China. Over the past 20 years, the government has been committed to improving rural feces and sewage pollution and strengthening the prevention and control of agricultural nonpoint source pollution. According to data from regional statistical yearbooks, the access rate to harmless sanitary toilets in rural China increased from 24.17% in 2002 to 62.5% in 2017. From 2003 to 2019, the total discharge of chemical oxygen demand (COD) and ammoniacal nitrogen in domestic sewage decreased by 42.76% and 52.76%, respectively (see Figure 2).

**Figure 3:**
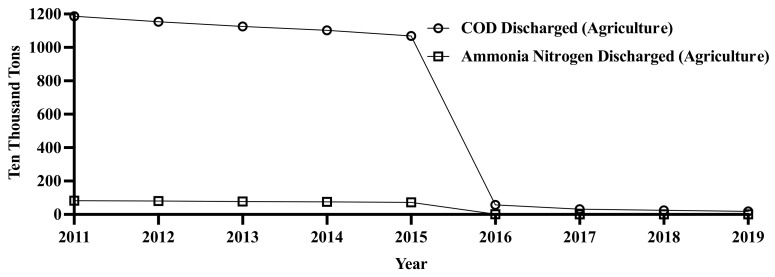
Discharge of agricultural wastewater. Source: China Environmental Statistical Yearbook (2011–2019).

With the implementation of the “National Plan for Sustainable Agricultural Development (2015–2030)” and the “General Plan for Addressing Prominent Agricultural Environment Problems (2014–2018),” in 2016, the Ministry of Agriculture of China issued the “Construction Plan of Demonstration Project for Comprehensive Control of Agricultural Nonpoint Source Pollution in Key River Basins (2016–2020),” highlighting that according to the report, long-term monitoring results from the National Agricultural Nonpoint Source Pollution Monitoring Network indicate that emissions of agricultural nonpoint source pollutants increased from 2007 to 2013, COD began to decline and total nitrogen stabilized, but the total emissions were still large. The report calls for stricter controls on agricultural water pollution to ensure that the water used for irrigation meets the required standards. This has resulted in remarkable results, with the COD for agricultural wastewater and total nitrogen emissions exhibiting a significant drop after 2016 (see Figure 3).

Specifically, this study explores the incidence and trend of zoonoses in various provinces of China, as well as its correlation with environmental health and economic development. Countermeasures and suggestions that can promote the long-term sustainable development of human, animal, and environmental systems are then proposed, which is rare in similar research literature.

## 2. Methodology

### 2.1. Measurement and Sample Selection

The definitions and measurements of the variables are shown below in Table 1.

Dependent variable: Among the eight zoonoses presented in Figure 1, brucellosis, malaria, and epidemic hemorrhagic fever have the highest average incidence. Compared with the other two zoonoses, the incidence of malaria is found to fluctuate significantly, although it exhibits a generally decreasing trend. Therefore, the incidence of malaria is selected as a consideration factor of human health, that is, as the dependent variable. The morbidity statistics for zoonoses are derived from Class A and B notifiable infectious diseases in the “China Health Statistics Yearbook”.

Independent variables: “Pesticide use” was selected as one of the independent variables. Before 2003, there were no statistics on access rate to harmless sanitary toilets, only sanitary feces disposal rate. This is because before 2003, rural fecal waste treatment measures focused on the popularization of sanitary toilets. With social and economic development, rural sanitation facilities have been constantly improving. Therefore, since 2003, attention has been paid to the utilization rate of harmless sanitary toilets. For example, COD and ammoniacal nitrogen emissions from agricultural wastewater have only been included in the Statistical Yearbook since 2011. These changes and increases in the statistical caliber reflect the efforts and development of environmental governance in rural China over the past two decades; however, it creates a barrier to data continuity. Based on the above, the consumption of agricultural diesel oil and pesticides and investment in lavatory sanitation improvement in rural areas were selected as environmental factor indicators. Data on environmental factors were obtained from the “China Rural Statistical Yearbook” and “China Environmental Statistical Yearbook”.

Control variable: The health level and living environment in rural areas are affected by the level of economic and social development, and the factors of rural economy, environment, and population interact closely with each other [21,26]. Therefore, considering the scientific and reasonable selection of indicators and availability of data, economic growth was chosen as the control variable. With the development of the rural economy, farmers’ incomes have increased significantly, and the area of economic activities has gradually expanded. In this study, farmers’ per capita disposable income was selected to measure the improvement of farmers’ income level. Average temperatures and annual precipitation were selected as the control variables of climate factors [52,53]. The climate data came from the data sharing platform of the China Meteorological Administration (www.cma.gov.cn, accessed on 17 February 2022).

### 2.2. Model Construction

After adjusting for missing data (Tibet), the final sample consisted of 30 provinces and 390 province-year observations (Hong Kong, Macao, and Taiwan were not included). Panel data containing human health, environmental health, and economic growth factors of the sample spanned the period from 2005 to 2017.

Based on the above points, the benchmark measurement model of fixed effects is established:

(1) $$Malaria\;incidence_{it}=\alpha_{0}+\alpha_{1}Diesel\;fuel_{it}+\alpha_{2}Pesticide_{it}+\alpha_{3}Toilets\;investment_{it}+\alpha_{4}Income_{it}+\;\alpha_{5}Temperature_{it}+\alpha_{6}Precipitation_{it}+\varepsilon_{it}$$

where *i* represents the province, *t* represents the year, $Malaria\;incidence_{it}$ is measured by the number of cases per 100,000 population in the province, which is the dependent variable. $Diesel\;fuel_{it}$ (measured by the total agricultural consumption of diesel fuel in the province), $Pesticide_{it}$ (the total amount of pesticides used in the province), and $Toilets\;investment_{it}$ (the amount of investment in rural toilet improvement in the province) are independent variables. $Income_{it}$ (disposable income of rural residents in the province), $Temperature_{it}$ (measured by the mean of the average monthly temperatures), and $Precipitation_{it}$ (annual rainfall) are the control variables, $\varepsilon_{it}$ is the random interference term.

## 3. Results

**Figure 4:**
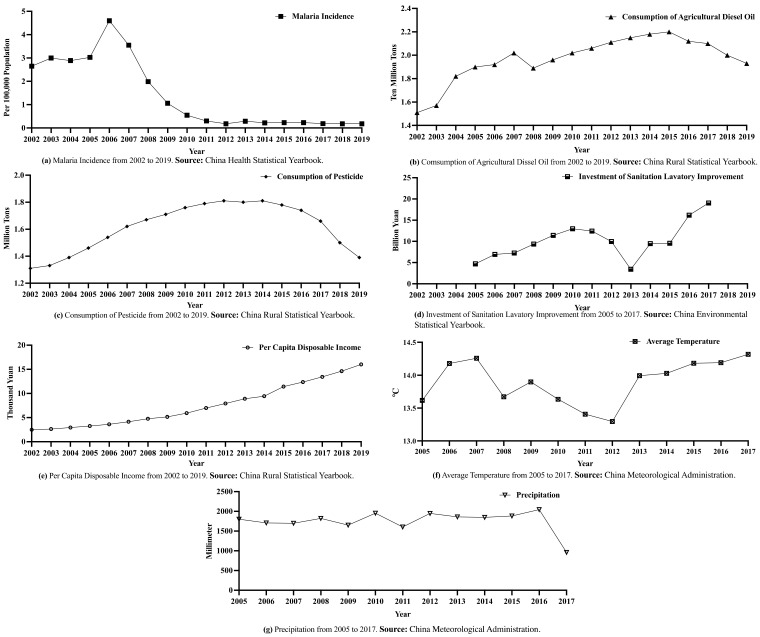
Malaria incidence with agricultural use of diesel and pesticides, investment in lavatory sanitation improvement, per capita disposable income of farmers, average temperature, and precipitation.

The incidence of malaria in relation to the consumption of diesel oil and pesticides, investment in rural toilet improvement, and per capita disposable income of farmers are illustrated in Figure 4. As shown, malaria incidence in 2006 was an inflection point, experiencing a rapid increase and then a significant decrease, after which it remained relatively flat and continued to decline slowly. In 2013, there was a major drop in investment in rural toilet improvement, which may correspond to the national fiscal revenue and expenditure released by the Ministry of Finance in 2013; data indicate that economic downturn, fiscal revenue reduction, and difficulties in achieving revenue budget occurred in 2013. With the gradual economic recovery and relevant measures to increase income, rural toilet improvement investment has experienced rapid growth and continues to receive attention. In 2019, the government continued to increase investment in and promote the improvement of the rural living environment, allocating 7 billion yuan of financial funds to implement the policy of awards and subsidies for the transformation of rural toilet facilities in entire villages. The fluctuation range of the average temperature was relatively stable, with the lowest temperature in 2012, and then the trend resumed as before. In general, from 2013 to 2017, the temperature increased slightly year by year. Precipitation is also relatively stable, with a sharp drop in 2017.

The descriptive statistics and correlations matrix of this study are shown in Table 2 and Table 3. As previously mentioned, the fixed effects regression model was employed to verify the relationship between malaria incidence and environmental health (agricultural use of diesel and pesticides, investment in lavatory sanitation improvement) with per capita disposable income of farmers average temperature and precipitation as control variables.

The empirical results are reported in Table 4. The consumption of pesticides has a negative influence on the malaria incidence with a *p*-value of 1%, while the investment in lavatory sanitation improvement and per capita disposable income have negative influences with a *p*-value of 5%. To control for the size effect, investment in lavatory sanitation improvement was measured by the logarithm of the amount of investment in rural toilet improvement. Average temperature has a positive relationship with the incidence of malaria. However, the precipitation is not significant.

## 4. Discussion

Previous research has highlighted a negative link between human health and environmental deterioration [54,55]. In particular, environmental degradation is one of the complex factors that causes an increase in outbreaks of disease [56]. The majority of previous studies have concentrated on understanding and controlling the negative impact of environmental decline on human health. This study therefore proposed suggestions targeting the improvement of the prevention of environmental pollution. The empirical results of the current study verified that there is not always a positive causal relationship between human health and environmental pollution. This result is consistent with Pan et al. [57], who found that the use of pesticides has an inhibiting effect on the spread of malaria. Their research revealed that changes in farming systems and the use of high doses of chemical pesticides in rice fields can to some extent destroy the breeding environment of Anopheles mosquitoes, reducing their distribution, number, or even leading to their “disappearance”, and weakening their malaria transmission effect. As highlighted by Wallace [31], the occurrence of zoonosis is associated with a wider range of fields other than public health and interacts with politics, social relations, and ecology. Therefore, understanding the occurrence and prevention of zoonosis needs to avoid preconceived causal assumptions and adopt new thinking and patterns to cope with such phenomena.

In terms of specific countermeasures, it is suggested to move away from mutually independent performance appraisal and focus on the integrated governance of humans, animals, and the environment. Further, establish a data exchange mechanism for human medicine, veterinary medicine, public health, environmental health, and other departments, and form a more complex analysis system of sector integration to avoid the dilemma of focusing on one and losing the other, thus having a more accurate and comprehensive understanding of the causal relationship between zoonosis and the environment. To realize effective long-term development, methodological approaches and cooperation techniques among sectors should be explored.

## 5. Conclusions

Based on the One Health concept, this study adopted panel data consisting of 30 provinces and 390 province–year observations (Tibet, Hong Kong, Macao, and Taiwan were not included) from 2005 to 2017 to explore the complex causal relationship between human and environmental health. The incidence of malaria was selected as the dependent variable, and the consumption of agricultural diesel oil and pesticides and investment in lavatory sanitation improvement in rural areas were selected as independent variables according to the characteristics of nonpoint source pollution and domestic pollution in China’s rural areas. By employing a fixed effects regression model, the empirical results show that the use of pesticides is negatively correlated with the incidence of malaria, implying a link between pesticide use and malaria. Continuous investment in rural toilet improvement and an increase in economic income can play a positive role in the prevention and control of malaria. Results also show that higher average temperatures are associated with higher incidence of malaria. The findings of this study on the influence of precipitation on malaria incidence are not significant, indicating that rainfall is not necessarily a determinant of malaria incidence [58,59].

This study presents an ecological analysis of malaria trends and a series of environmental indicators. It has the following two limitations. First, malaria is not the only zoonotic disease that threatens human health in rural areas. Although this study selected the most revealing disease, its health indicators are relatively single. Secondly, the incidence of malaria is influenced by many factors, such as humidity, local public health, and the level of government attention, all of which can affect the prevalence of malaria. Future research could be directed towards calculating a composite epidemic index to represent health levels based on the incidence of major zoonoses in different regions, while incorporating more comprehensive determinants to take into account complex causal relationships. In addition, this study focuses on zoonoses as a health indicator because it can better reflect the intersection of human health and animal health in the One Health concept. However, there is no doubt that the impact of human activities on animal and environmental health is far more than this. In addition to animal-borne infectious diseases, other diseases, such as cancer and chronic diseases, also have a complex relationship with the environment and animal health. This is the direction this study will continue to explore in future research.

## Figures and Tables

**Table 1:** One Health factors and variables.

Factors	Name	Unit	Description
Human health factor	Malaria incidence	1/100,000	No. of cases per 100,000 population in the region
Environmental health factor	Diesel fuel	10,000 tons	Total agricultural consumption of diesel fuel in the region
	Pesticide	ton	Total amount of pesticides used in the region
	Toilets investment	100,000 Yuan	Amount of investment in rural toilet improvement in the region
Economic growth factor	Income	Yuan/per	Disposable income of rural residents in the region
Climate factor	Average temperature	°C	The mean of the average monthly temperatures.
	Precipitation	Millimeter	Annual rainfall

**Table 2:** Descriptive statistics.

Variables	Min.	Max.	Mean	Standard Deviation	Coefficient of Variation	Skewness	Kurtosis
Malaria incidence	0	57.16	1.34	6.18	4.61	49.74	6.65
Consumption of agricultural diesel oil	2.20	487.00	68.14	67.97	1.00	12.22	2.56
Consumption of pesticide	1599	173,461	56,735.37	43,768.41	0.77	2.36	0.54
Investment of sanitation lavatory improvement	28.60	419,744	35,474.76	43,186.88	1.22	30.48	4.18
Per capital disposable income	1876.96	27,825	7995.81	4639.12	0.58	4.96	1.28
Average temperature	2.55	25.13	13.90	5.35	0.38	2.40	−0.21
Precipitation	55.1	5111.30	1751.81	1251.21	0.71	2.80	0.72

**Table 3:** Correlations matrix.

Variables	1.	2.	3.	4.	5.	6.
1. Malaria incidence	1					
2. Consumption of agricultural diesel oil	−0.0682	1				
3. Consumption of pesticide	0.0132	0.4686 **	1			
4. Investment of sanitation lavatory improvement	−0.0763	0.2530 **	0.3818 **	1		
5. Per capital disposable income	−0.1911 **	0.0801	−0.0233	0.1542 **	1	
6. Average temperature	0.2132 **	−0.0366	0.2925 **	0.2918 **	0.1443 **	1
7. Precipitation	0.0126	0.0094	0.2506 **	0.2282 **	−0.2589 **	0.1794 **

** *p* < 0.01.

**Table 4:** Results of fixed effects regression model.

Variables	Malaria Incidence (1/100,000)
Constant	1.0259
	(0.11)
Consumption of agricultural diesel oil	−0.0063
	(−0.40)
Consumption of pesticide	−0.0002 **
	(−4.43)
Investment of sanitation lavatory improvement	−1.6078 *
	(−2.27)
Per capital disposable income	−0.0003 **
	(−3.69)
Average temperature	1.3844 *
	(2.20)
Precipitation	−0.0001
	(−0.26)
Year	Control
Province	Control
Number of groups	30
Number of observations	390
F-value	9.96

** *p* < 0.01, * *p* < 0.05.

## Data Availability

The data presented in this study are available on request from the corresponding author. The data are not publicly available due to [Data used in the study was obtained through account applications. Restrictions apply to the availability of these data and are therefore not publicly available. However, data is available from the authors upon reasonable request].
